# Identification of Key Genes and Molecular Pathways in Keratoconus: Integrating Text Mining and Bioinformatics Analysis

**DOI:** 10.1155/2022/4740141

**Published:** 2022-08-23

**Authors:** Di Hu, Zenan Lin, Junhong Jiang, Pan Li, Zhehuan Zhang, Chenhao Yang

**Affiliations:** ^1^Department of Ophthalmology, Children's Hospital of Fudan University, National Children's Medical Center, Shanghai 201102, China; ^2^Department of Ophthalmology, Shanghai General Hospital, Shanghai Jiao Tong University School of Medicine, Shanghai, China; ^3^Department of Ophthalmology, First Hospital of Xi'an, Institute of Ophthalmology, Key Lab of Ophthalmology, Clinical Center for Ophthalmology, Xi'an 710002, China

## Abstract

**Purpose:**

To identify the potential key genes and molecular pathways associated with keratoconus and allergic disease.

**Methods:**

The pubmed2ensembl database was used to identify the text mining genes (TMGs) collectively involved in keratoconus and allergic disease. The GeneCodis program was used to perform the Gene Ontology (GO) biological process and Kyoto Encyclopedia of Genes and Genomes (KEGG) pathway enrichment analysis of TMGs. The protein-protein interaction (PPI) network of the TMGs was established by STRING; the significant gene modules and hub genes of PPI were further performed using the Cytoscape software. The DAVID database was used to perform the GO and KEGG analyses of the significant module.

**Results:**

In total, 98 TMGs collectively involved in keratoconus and allergic disease were identified. 19 enriched biological processes including 71 genes and 25 enriched KEGG pathways including 59 genes were obtained. A TMG PPI network was constructed, and 51 genes/nodes were identified with 110 edges; 3 most significant modules and 12 hub genes were chosen from the PPIs. GO enrichment analysis showed that the TMGs were primarily associated with collagen catabolic process, extracellular matrix organization and disassembly, cell adhesion and migration, collagen-containing extracellular matrix, extracellular matrix, and structure organization. KEGG pathway analysis showed that these DEGs were mainly involved in the IL-17 signaling pathway, inflammatory bowel disease, rheumatoid arthritis, allograft rejection, T cell receptor signaling pathway, cytokine-cytokine receptor interaction, and TNF signaling pathway.

**Conclusions:**

The results revealed that *IL10*, *IL6*, *MMP9*, *MMP1*, *HGF*, *VEGFA*, *MMP3*, *MMP2*, *TGFB1*, *IL4*, *IL2*, and *IFNG* were potential key genes involved in keratoconus. IL-17 signaling pathway was the potential pathways accounting for pathogenesis and development of keratoconus.

## 1. Introduction

Keratoconus (KC) is a corneal ectasia disease characterized by thinning and steepening, which would cause irregular astigmatism and progressive myopia, leading to further loss of vision [1]. KC is considered to be a relatively rare disease in the past. However, with advances in diagnostic devices technology, an increasing number of patients with KC are being diagnosed [2]. The global prevalence of KC was 138 per 100000, and it has become one of the most common degenerative corneal diseases [3]. KC typically has its onset in the adolescent and progresses until the third or fourth decade of life [4, 5]. Therefore, KC has now become one of the most common causes leading to visual impairment in adolescent population, resulting heavy economic burden to individuals and society.

At present, various treatments for KC exist, including spectacles, contact lenses, corneal collagen cross-linking (CXL), and corneal surgery. However, these treatments present several limitations. In its early stages, spectacles or rigid contact lenses only improve the visual impairment but cannot delay or prevent the degeneration of KC [6]. CXL can increase the stiffness of the cornea and slow the progression of moderate KC, but it is still accompanied by complications including epithelial damage, keratitis, and endothelial damage [7–9]. For advanced KC, keratoplasty is the preferred therapeutic strategy; however, the shortage of donor organs, immune rejection after transplantation, and graft infection limit its application [10]. Clearly, a more effective therapy for the treatment and prevention of KC is urgently needed.

KC is a multifactorial disease that involves several genes and environmental factors [11]. Allergic disease is one of the major risk factors for KC. A relationship between KC and allergic disease was reported by Hilgartner et al. as early as in the 1937 [12]. Subsequent studies established that there is a positive association between allergic disease and KC, with a prevalence reaching 11% to 30% [13–15]. In a recent meta-analysis of 29 studies from 15 countries with 7 million participants, the odds ratio (OR) of developing KC was 1.42 times higher in subjects with allergic disease versus healthy subjects [3]. A 2021 nationwide study in the Netherlands found a statistically significant positive association between KC and allergic diseases, which include allergic rash (OR = 3.00), asthma and bronchial hyperresponsiveness (OR = 2.51), and allergic rhinitis (OR = 2.20) [16]. Additionally, allergic eye disease is also considered to be closely associated with KC. KC patients with vernal keratoconjunctivitis or allergic conjunctivitis tend to have significantly thinner and steeper corneas [17, 18].

Corneal stroma, which consists of keratocytes and extracellular matrix (ECM), is the main structural fraction of the cornea accounting for 90% of corneal thickness [19]. And the progressive thinning of corneal stroma is the primary structural changes in KC [20]. The remodeling of ECM would influence the biomechanical properties of corneal stroma and consequently involved in the development and progression of KC [21–25]. Previous studies have demonstrated that risk factor of KC including eye rubbing and contact lens wearing can trigger the remodeling of ECM through upregulation of matrix metalloproteinases (MMPs), which are the primary regulators of ECM remodeling [26], while the upregulation of MMP expression has been also observed in patients with allergic diseases, indicating that allergic diseases may contribute to KC by promoting ECM remodeling [27]. Therefore, allergic diseases might be a potential target for the prevention and treatment of KC.

Text mining, an effective method to quickly extract critical information from a large amount of the biomedical literature, has been widely used to explore novel associations between genes and pathologies [28]. In recent years, biomarkers are widely applied to accurate diagnosis and personalized treatment of diseases. Information technology can speed up screening process of biomarkers. The in-depth study on the omics cascade of KC laid the foundation for bioinformatic analysis. The aim of the present study was to explore the key genes and molecular pathways associated with KC and allergic disease, by integrating text mining and bioinformatics analysis.

## 2. Methods

### 2.1. Data Collection

The pubmed2ensembl website (http://pubmed2ensembl.ls.manchester.ac.uk/) [28] is an online database resource that links over 2,000,000 articles in PubMed to approximately 150,000 genes in Ensembl from 50 species. In order to identify the common genes involved in keratoconus and allergic disease, we perform the text mining using pubmed2ensembl. In detail, we determined the two queries with the terms “keratoconus” and “allergic disease,” in the species dataset of “Homo sapiens (GRCh37).” The queries returned two lists of genes; the unduplicated genes were extracted and the intersection of which was then used as the text mining genes (TMGs).

### 2.2. Functional Enrichment Analysis of TMGs

The GeneCodis [29] was used to perform functional enrichment analysis of TMGs related to keratoconus and allergic disease. The Gene Ontology (GO) biological process annotations of the TMGs were analyzed, and genes with significantly enriched biological processes were selected and used for further analysis of enriched Kyoto Encyclopedia of Genes and Genomes (KEGG) pathway annotations. Genes in the significant enriched KEGG pathways were selected for further analysis. A corrected *P* value cut-off (*P* = 0.00001) was set.

### 2.3. Protein-Protein Interaction (PPI) Network

In order to assess functional associations among the products of the selected TMGs, the STRING (version 11.5, https://string-db.org/), a database includes 3 billion interactions associated with 24.6 million proteins referred to 5090 organs [30], was used to construct the PPI network. “Homo sapiens” was selected as the species dataset, and the highest confidence score (0.900) was set as the minimum required interaction score.

### 2.4. Module Analysis and Hub Gene Identification

The Cytoscape software (version 3.9.1) was used to visualize the PPI network [31]. Then, the significant gene modules in the PPI networks were identified using the Molecular Complex Detection (MCODE) plugin of Cytoscape [32]. The standard settings of MCODE rest were as follows: degree cutoff = 2, node score cutoff = 0.2, *k*‐core = 2, and maximum depth = 100.

Moreover, to select hub genes from the PPI network, the cytoHubba plugin of Cytoscape was used, which was computed by four ranking methods: EPC (edge percolated component), MCC (maximal clique centrality), MNC (maximal neighborhood component), and DMNC (density of maximum neighborhood component) [33]. The top 15 genes within the four methods were screened and overlapped, and the overlapping genes were considered to be hub genes.

### 2.5. Functional Enrichment Analyses of Module and Hub Gene

To clarify the functions of the significant genes module, the DAVID database (https://david.ncifcrf.gov/) was used to perform GO enrichment analysis and KEGG pathway enrichment analysis. The GO enrichment analysis includes biological process (BP), cellular component (CC), and molecular function (MF) [29]. The functional enrichment analyses of the hub genes were performed and visualized using the Cytoscape plugins ClueGO (version 2.5.7) and CluePedia (version 1.5.7) [31]. *P* < 0.05 was considered statistically significant.

## 3. Results

### 3.1. Identification of TMGs

**Figure 1:**
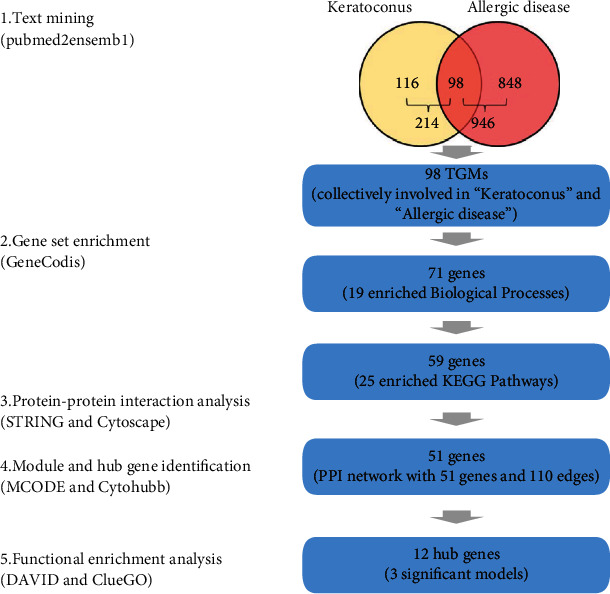
Overall strategy used for the identification of potential key genes and molecular pathways associated with keratoconus and allergic disease.

Based on the data mining strategy that is described in Figure 1, 946 unique genes related to allergic disease and 214 unique genes related to keratoconus were acquired through text mining searches. There were 98 genes common to the 2 gene lists (Supplementary Table 1).

### 3.2. Functional Enrichment Analysis of TMGs

The 98 TMGs were analyzed for GO biological processes (BP) using GeneCodis to identify the most enriched terms closely related to the pathology of keratoconus. As shown in Table 1, 19 significantly enriched GO annotations of 71 unique genes were identified. The top 10 BPs are associated with positive regulation of gene expression, positive regulation of phosphatidylinositol 3-kinase signaling, response to hypoxia, positive regulation of MAPK cascade, collagen catabolic process, immune response, inflammatory response, extracellular matrix disassembly, response to lipopolysaccharide, and cellular response to ultraviolet-A (UV-A).

Next, the 71 TMGs were further analyzed for KEGG pathways using GeneCodis. The KEGG pathway enrichment analysis identified 25 significant pathways that involved 59 TMGs. The top 10 KEGG pathway contained malaria, allograft rejection, rheumatoid arthritis, cytokine-cytokine receptor interaction, pathways in cancer, AGE-RAGE signaling pathway in diabetic complications, graft-versus-host disease, type I diabetes mellitus, and inflammatory bowel disease (Table 2).

### 3.3. PPI Network Construction, Modular Analysis, and Hub Gene Identification

**Figure 2:**
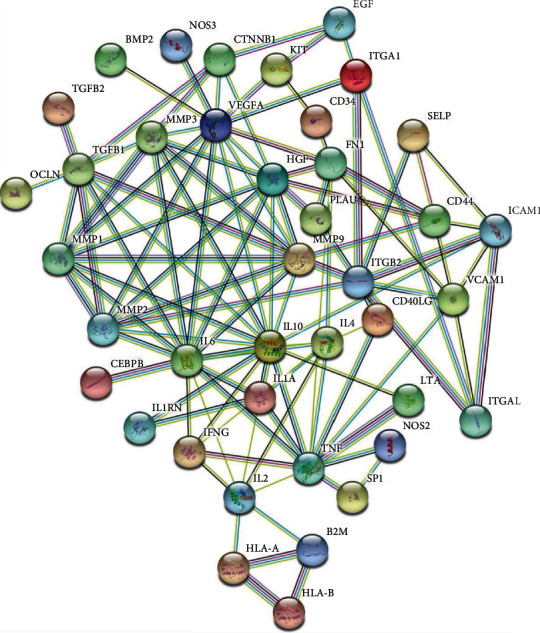
The protein–protein interaction network of the 59 target TMGs.

**Figure 3:**
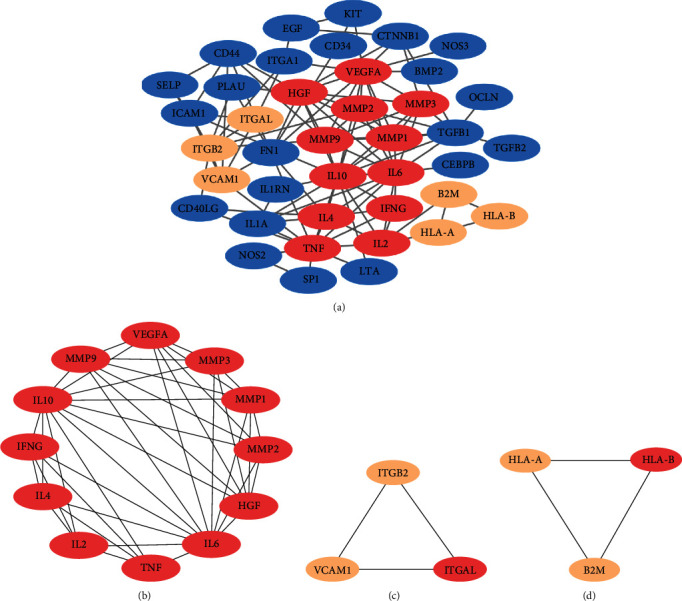
(a) The protein–protein interaction (PPI) network of the target TMGs was visualized using Cytoscape. (b–d) The three modules were obtained from PPI network using MCODE: (b) module 1; (c) module 2; (d) module 3.

The PPI network of the 59 selected TMGs was constructed using the STRING database, which had a total of 51 nodes with 110 edges (Figure 2). Furthermore, a subnetwork clustering analysis was performed using the MCODE plugin in Cytoscape, and three modules were selected as the significant modules in the PPI network (Figure 3(a)). Module A included 12 genes/nodes and 41 edges, module B included 3 genes/nodes and 3 edges, and module C included 3 genes/nodes and 3 edges (Figures 3(b)–3(d)).

**Figure 4:**
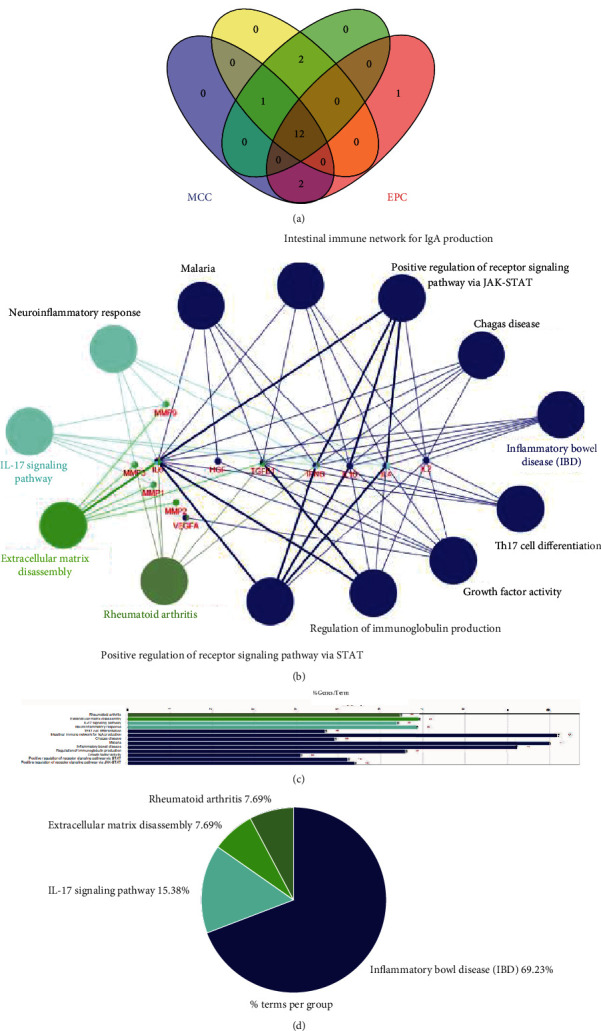
(a) Overlapping TMGs among the four topological cytoHubba methods including MCC, MNC, DMNC, and EPC. (b) Functions and pathways of the hub genes were visualized using ClueGO. (c) Enriched GO terms and KEGG pathways. (d) Distribution of the functions and pathways among the hub genes. Only the most significant term in the group was labeled. Representative enriched pathway (*P* < 0.05) interactions among the hub genes.

The top 15 ranking hub genes were identified by the DMNC, MNC, MCC, and EPC methods using the cytoHubba plugin, and the 12 overlapping genes, including *IL10*, *IL6*, *MMP9*, *MMP1*, *HGF*, *VEGFA*, *MMP3*, *MMP2*, *TGFB1*, *IL4*, *IL2*, and *IFNG*, were considered to be hub genes (Figure 4(a)).

### 3.4. Functional Enrichment Analyses of Module and Hub Gene

**Figure 5:**
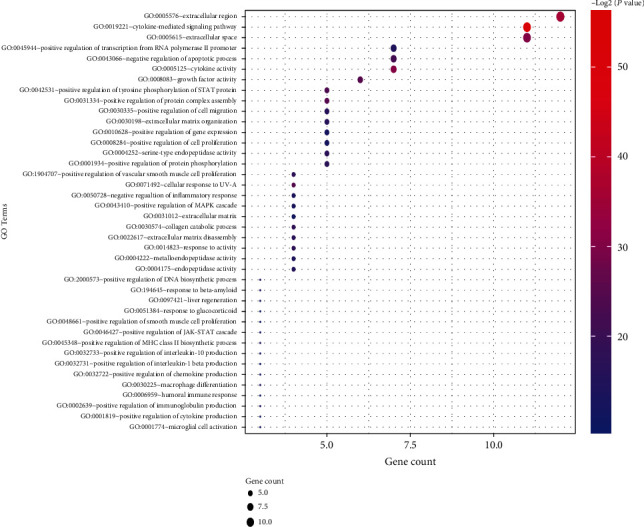
Top 40 significantly enriched GO terms in module 1.

**Figure 6:**
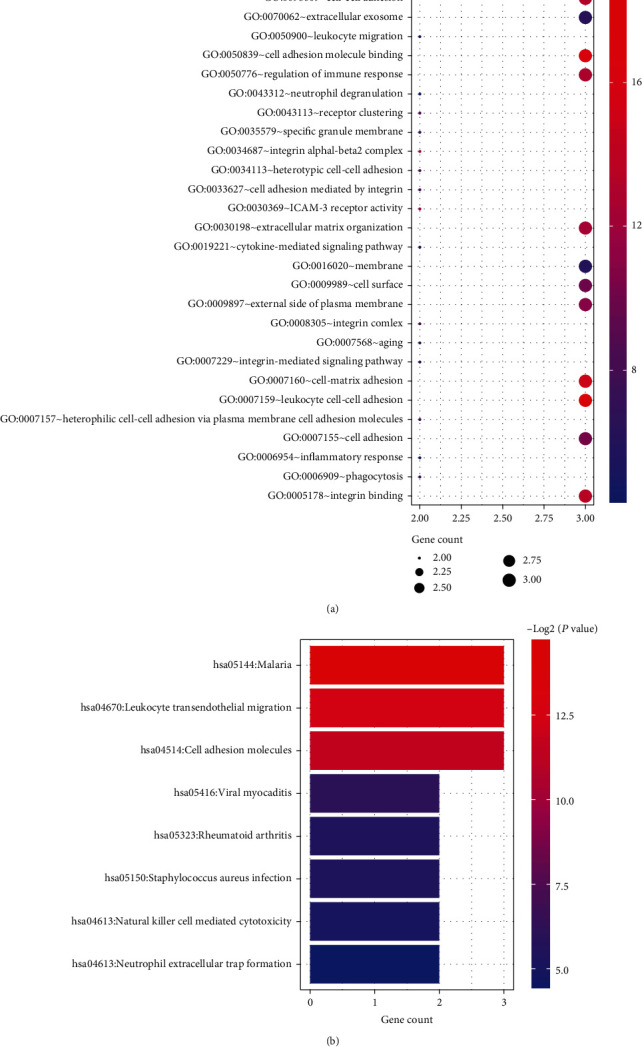
(a) The significantly enriched GO terms in module 2. (b) The significantly enriched KEGG pathways in module 2.

**Figure 7:**
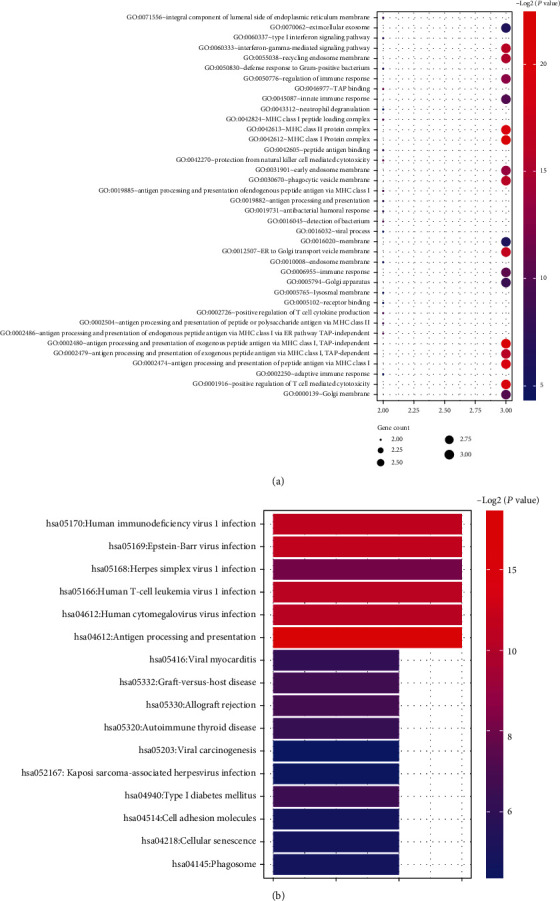
(a) The significantly enriched GO terms in module 3. (b) The significantly enriched KEGG pathways in module 3.

The GO and KEGG pathway enrichment analyses of significant modules were performed by DAVID database. As shown in Figure 5, the GO enrichment analysis showed that the genes in module 1 were mainly enriched in the biological processes associated with ECM remodeling (collagen catabolic process, extracellular matrix organization, and disassembly), immune inflammatory response (negative regulation of inflammatory response, positive regulation of immunoglobulin production, humoral immune response, type 2 immune response, negative regulation of cytokine production involved in immune response, etc.), and response to stimuli (response to hypoxia, UV-A, beta-amyloid, glucocorticoid, and xenobiotic stimulus). Module 2 was enriched in the biological processes associated with extracellular matrix organization, cell adhesion and migration, and immune and inflammatory response (Figure 6(a)). Module 3 was enriched in immune inflammatory response (Figure 7(a)).

**Figure 8:**
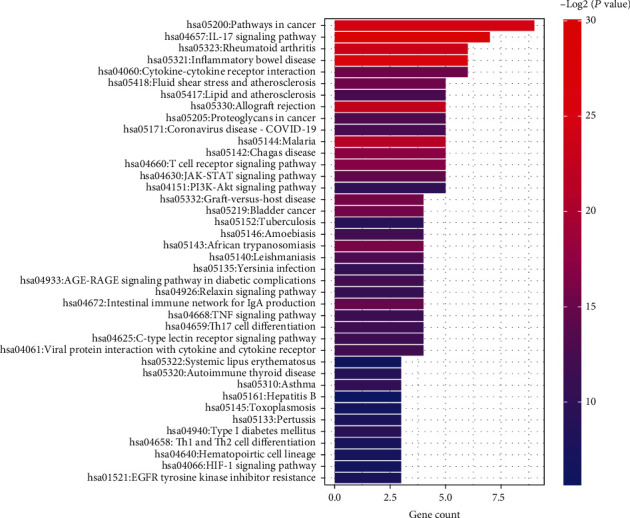
Top 40 significantly enriched KEGG pathways in module 1.

KEGG pathway analysis revealed that the module 1 was mainly enriched during IL-17 signaling pathway, inflammatory bowel disease, rheumatoid arthritis, allograft rejection, T cell receptor signaling pathway, cytokine-cytokine receptor interaction, intestinal immune network for IgA production, JAK-STAT signaling pathway, TNF signaling pathway, etc. (Figure 8). Module 2 was mainly enriched in leukocyte transendothelial migration, cell adhesion molecules, rheumatoid arthritis, natural killer cell-mediated cytotoxicity, etc. (Figure 6(b)). Module 3 was mainly enriched in virus infection, autoimmune thyroid disease, allograft rejection, and antigen processing and presentation (Figure 7(b)).

The functional enrichment analysis of 12 hub genes was performed using the ClueGO and CluePedia in Cytoscape. As shown in Figure 4(b), these hub genes were mainly enriched in terms of rheumatoid arthritis, extracellular matrix disassembly, inflammatory bowel disease, IL-17 signaling pathway, and regulation of immunoglobulin production (Figures 4(c) and 4(d)).

## 4. Discussion

Keratoconus is a multifactorial corneal disorder characterized by progressive thinning of the corneal tissue, which can lead to severe visual impairment. Although many etiology studies have been conducted, the exact pathogenesis of KC is still poorly understood [1]. Allergic diseases are the risk factor for the development and progression of KC. In this study, our purposes were to explore the key genes and molecular pathways of KC through determining the genes and molecular pathways associated with keratoconus and allergic disease. Firstly, 214 unique genes related to keratoconus and 1, 946 genes related to allergic disease were acquired via text mining, and 98 TMGs collectively involved in keratoconus and allergic disease were identified. Furthermore, we investigated the biological functions of these TMGs, 19 significantly enriched GO-BP annotations including 71 genes were identified via GO analysis, and 25 enriched KEGG pathways including 59 genes were identified via KEGG pathway analysis. Additionally, a TMG PPI network was constructed, and 51 genes/nodes were identified with 110 edges, and 3 most significant modules were chosen from the PPIs. Finally, 12 hub genes, *IL10*, *IL6*, *MMP9*, *MMP1*, *HGF*, *VEGFA*, *MMP3*, *MMP2*, *TGFB1*, *IL4*, *IL2*, and *IFNG*, were identified.

Keratoconus is known to be closely associated with allergic diseases, and the reported prevalence ranges from 11 to 30% [34]. Woodward et al. evaluated 16053 keratoconus patients in America and found a significant association between KC with allergic disease [35]. The same conclusion was also obtained in another study of 807 KC patients compared to 600,000 controls in Israel [36]. A recent meta-analysis reviewed 29 articles and included over 7158241 people from 15 countries, indicating that people with allergy were more likely to having keratoconus, with odds ratios of 1.42 (95% CI: 1.06–1.79) [3]. Merdler et al. found a significant association between KC and allergic conjunctivitis, chronic blepharitis, vernal keratoconjunctivitis, asthma, and allergic rhinitis [37]. However, the exact relationship between KC and allergic diseases has remained elusive. In the present study, GO enrichment analysis showed that the TMGs were significantly enriched in immune inflammatory response-related terms including regulation of MHC class II biosynthetic process, immune response, immunoglobulin production and T cell-mediated cytotoxicity, antigen processing and presentation, and regulation of inflammatory response. Immune inflammatory response is one of the key links of allergic diseases [38]. These findings suggest that allergic diseases may participate in the development of KC through the immune inflammatory mechanisms.

In the present study, the enriched GO biological process analyses showed that the TMGs were associated mainly with collagen catabolic process, extracellular matrix organization and disassembly, cell adhesion, and migration which play crucial role in corneal ECM remodeling. The cornea is composed of five layers, with the corneal stroma being the main structural fraction, accounting for 90% of corneal thickness [19]. Extracellular matrix (ECM), which is made up of collagen, laminins, and fibronectins, is the predominant component of the cornea stroma [39]. The alterations in the composition or structure of corneal ECM are recognized as critical in the pathogenesis and progression of KC [22]. An updated proteomic study has also confirmed that the structural collagen expression decreased broadly in patients with KC [24], which is consistent with our study. Results of the present study suggested that allergic disease may be involved in the initiation and development of KC by regulating the ECM remodeling of corneal stroma. Matrix metalloproteinase (MMP) family is a zinc-dependent endopeptidase family that can degrade the components of ECM [40, 41]. And MMP is expressed at a high level in tears and corneal tissues of patients compared with allergic diseases [42–44]. Thus, it is assumed that allergic diseases may contribute to KC by promoting ECM remodeling.

In the present study, the enriched KEGG pathway analysis revealed that TMGs were associated mainly with IL-17 signaling pathway. Interleukin-17 (IL-17) family, which is composed of IL-17A–F, plays a crucial role in acute and chronic inflammatory reactions [45]. IL-17 has been shown to promote IL-6 and IL-8 release which can lead to an acute phase response such as a fever and the accumulation of neutrophils. In addition, IL-17 activates the function and production of MMPs during chronic inflammation [46]. IL-17 family signals bind and signal via the IL-17 receptor, activating multiple downstream pathways such as NF*κ*B, MAPKs, and C/EBPs [47]. Gomes et al. reported that the IL-17 polymorphism was related with KC [48]. Karolak et al. sequenced an Ecuadorian family with KC and confirmed that the c.527G4A in IL-17B is variant in KC [49]. The proteomic analysis of tears revealed the upregulation of IL-17 in KC patients, which may play an important role in the occurrence of KC by inducing the expression of IL-6 and IL-8 and activating the production of MMPs [50]. Therefore, IL-17 signaling pathway may be a potential key pathway involved in KC.

Interleukin-10 (IL-10) is an anti-inflammatory cytokine with important roles in preventing T helper type 1 cells from producing cytokine. Several studies have shown that there was no obvious change in IL-10 in tears of keratoconus and control subjects [51–53], while a few studies have suggested that there was reduced IL-10 in epithelium of KC patients [54]. Interleukin-6 (IL-6) is a multifunctional proinflammatory cytokine which plays an important role in numerous immune-mediated diseases. Previous studies demonstrated that tear level of IL-6 was significantly higher in patients with KC, and there was a significant positive correlation between the IL-6 level and the KC severity [55]. IL-6 can promote MMP production, leading to ECM of corneal stroma degradation [44]. The interleukin-2 and interleukin-7 (IL-2, IL-7) are important factors in regulating lymphoid development. IL-2 is a proinflammatory factor, which can promote the generation of antigen-specific immune reactions [56]. IL-4 is an anti-inflammatory cytokine which can promote Th2 differentiation [57, 58]. IL-2 and IL-4 are more highly expressed in patients with KC rather than in normal people [59]. The changes of IL-10, IL-6, IL-2, and IL-4 expressions indicated a modification of inflammatory environment in the pathogenesis of KC. MMP-1, MMP-2, MMP-3, and MMP-9 all are members of the MMP family which implicated in inflammation and degradation of the ECM components [40, 41]. Plenty of evidence suggest that MMP expressions are significantly increased in tears and corneal tissues in patients with KC, indicating that proteolytic dysregulation participates in the process of KC [26, 60, 61]. The MMP-1 expression levels were elevated in the corneal epithelium, stromal, and tears in patients with KC. MMP-1 can degrade Col I and III in cornea, resulting in stroma thinning in the onset and progression of KC [44, 62]. MMP-2 is a major secreted protease in the normal cornea tissue and plays an important role in degradation and remodeling of the corneal ECM, but with more conflicting results [63]. Smith and Easty previously reported high levels of MMP-2 in keratocytes in keratoconic [43]. However, other studies on corneal tissue and in tears detected no increase of MMP-2 level in KC [44, 64]. Among relationship between MMPs and KC, MMP-9 is the most studied one. Multiple studies have shown that MMP-9 is increased in tears, cells from the cone apex, and blood of KC patients [42, 54, 65, 66]. Several studies have revealed that MMP-9 in tears is an early diagnostic marker of KC [42, 65, 67, 68]. The MMP-3 expression was also found to increase in tears of KC patients [44]. Interestingly, in cultures of keratoconic in vitro, the expression of MMP-1, MMP-2, MMP-3, and MMP-9 is decreased after CXL treatment [69, 70]. Hepatocyte growth factor (HGF) is a multifunctional growth factor, which was defined as the growth factor of fibroblast-derived cell [71]. Recent case-control studies suggested that variant of HGF is a candidate risk factor of KC [72, 73]. TGF-*β* includes three isoforms in mammals, TGF-*β*1, TGF-*β*2, and TGF-*β*3, and is a key mediator of fibrogenesis [74]. A recent study demonstrated increased TGF-*β* markers in severe keratoconus patients [75]. It was reported that TGF*β* contributes to upregulate the expression of MMP2 by modulating Smad2 [76]. Interferon gamma (IFNG) is a critical proinflammatory cytokine which can regulate immune system [77]. It was proved that IFNG can negatively regulate the expression of TGF-*β*. These genes are the potential key genes that may be involved in KC.

Strengths of our study include the fact that it is one of the first to use text mining and bioinformatics analysis to identify the potential crucial genes and key pathways of KC based on the common genes involved in KC and allergic disease. Limitations of this study must also be acknowledged. Firstly, this study explored the molecular mechanism of keratoconus in the gene level using bioinformatics analysis; further experimental studies are required to verify the results. Secondly, adolescents' population is a high-risk group for KC, but we did not include the age factor in this research; future investigations taking the age into consideration may provide more accurate and comprehensive conclusions.

In conclusion, we identified 12 hub genes, *IL10*, *IL6*, *MMP9*, *MMP1*, *HGF*, *VEGFA*, *MMP3*, *MMP2*, *TGFB1*, *IL4*, *IL2*, and *IFNG*, that may be involved in the keratoconus as well as in allergic diseases. These genes were enriched in the HIF-1 signaling pathway, T cell receptor signaling pathway, and TNF signaling pathway. Extracellular matrix remodeling and immune inflammatory response may be the key alterations in KC. The absence of experimental validation is a limitation of this study, and further studies are needed.

## Figures and Tables

**Table 1:** Top nineteen GO biological processes of TMGs.

Process	Genes in query set	Total genes in genome	Corrected hypergeometric *P* value	Genes
Positive regulation of gene expression	24	516	6.21*E*-13	VIM, FN1, BMP2, MMP8, OCLN, CTNNB1, CEBPB, EDA, KIT, TGFB1, EGF, NGF, NOS3, SP1, CD34, GSN, IFNG, TNF, VEGFA, IL1A, IL6, TNC, ATM, IL4
Positive regulation of phosphatidylinositol 3-kinase signaling	11	81	5.39*E*-10	CAT, SELP, HGF, FN1, KIT, EGF, LEP, TNF, VEGFA, TGFB2, MYOC
Response to hypoxia	13	159	1.98*E*-09	CAT, BMP2, LEP, MMP2, TNF, VEGFA, TGFB2, LTA, IL1A, VCAM1, PLAU, ATM, NOS2
Positive regulation of MAPK cascade	13	172	4.05*E*-09	HGF, BMP2, MMP8, CTNNB1, KIT, EGF, LEP, ALK, ITGA1, TNF, VEGFA, IL6, SOD1
Collagen catabolic process	8	38	8.58*E*-09	MMP1, MMP8, MMP13, MMP10, MMP9, CTSB, MMP2, MMP3
Immune response	18	477	2.18*E*-08	IL10, HLA-B, MS4A2, CD40LG, IL1RN, CEBPB, EDA, FASLG, IL2, IFNG, IL16, HLA-A, TNF, LTA, IL1A, IL6, B2M, IL4
Inflammatory response	17	413	2.18*E*-08	SELP, ITGB2, BMP2, ITCH, LYZ, MS4A2, CD40LG, IL1RN, CEBPB, KIT, TGFB1, TNF, IL1A, IL6, ITGAL, CD44, NOS2
Extracellular matrix disassembly	8	48	3.96*E*-08	MMP1, MMP8, MMP13, MMP10, MMP9, GSN, MMP2, MMP3
Response to lipopolysaccharide	11	161	2.30*E*-07	SELP, IL10, CEBPB, FASLG, NOS3, TNF, LTA, BCR, IL1A, VCAM1, NOS2
Cellular response to UV-A	5	11	3.38*E*-07	MMP1, MMP9, MMP2, MMP3, TIMP1
Positive regulation of tyrosine phosphorylation of STAT protein	8	68	5.16*E*-07	KIT, LEP, IL2, IFNG, TNF, VEGFA, IL6, IL4
Negative regulation of apoptotic process	17	529	5.17*E*-07	CAT, FLNA, HGF, IL10, ITCH, CTNNB1, CD40LG, LEP, MMP9, IL2, TNF, VEGFA, IL6, CD44, SOD1, IL4, TIMP1
Cell-matrix adhesion	9	103	6.37*E*-07	ITGB2, FN1, CTNNB1, EDA, ITGA1, CD34, VCAM1, ITGAL, CD44
Leukocyte cell-cell adhesion	6	30	1.48*E*-06	SELP, ITGB2, CD40LG, ICAM1, VCAM1, ITGAL
Positive regulation of cell migration	12	259	1.92*E*-06	HGF, BMP2, KIT, TGFB1, EGF, MMP9, MMP2, VEGFA, PLAU, ATM, MYOC, IL4
Extracellular matrix organization	10	171	3.41*E*-06	MMP1, ITGB2, MMP8, MMP13, MMP10, MMP9, MMP2, TNF, MMP3, ITGAL
Positive regulation of cell population proliferation	16	548	4.23*E*-06	RPS4X, FN1, CTNNB1, KIT, TGFB1, FASLG, EGF, LEP, IL2, IFNG, VEGFA, TGFB2, IL6, TNC, IL4, TIMP1
Cellular response to lipopolysaccharide	10	182	5.47*E*-06	VIM, IL10, CD68, CEBPB, TNF, BCR, IL1A, IL6, NOS2, B2M
Positive regulation of interleukin-6 production	8	99	6.03*E*-06	MMP8, LEP, IFNG, IL16, TNF, IL1A, IL6, NOS2

**Table 2:** Top twenty-four KEGG pathways of TMGs.

Process	Genes in query set	Total genes in genome	Corrected hypergeometric *P* value	Genes
Malaria	13	50	6.57*E*-15	SELP, HGF, ITGB2, IL10, CD40LG, TGFB1, IFNG, TNF, ICAM1, TGFB2, IL6, VCAM1, ITGAL
Allograft rejection	9	37	3.17*E*-10	IL10, HLA-B, CD40LG, FASLG, IL2, IFNG, HLA-A, TNF, IL4
Rheumatoid arthritis	12	91	3.17*E*-10	MMP1, ITGB2, TGFB1, IFNG, TNF, ICAM1, VEGFA, MMP3, TGFB2, IL1A, IL6, ITGAL
Cytokine-cytokine receptor interaction	18	293	3.17*E*-10	IL10, BMP2, CD40LG, IL1RN, EDA, TGFB1, FASLG, NGF, LEP, IL2, IFNG, IL16, TNF, TGFB2, LTA, IL1A, IL6, IL4
Pathways in cancer	21	531	8.07*E*-09	MMP1, HGF, FN1, BMP2, CTNNB1, KIT, TGFB1, FASLG, EGF, MMP9, ALK, SP1, IL2, IFNG, MMP2, VEGFA, TGFB2, BCR, IL6, NOS2, IL4
AGE-RAGE signaling pathway in diabetic complications	11	100	8.07*E*-09	FN1, TGFB1, NOS3, MMP2, TNF, ICAM1, VEGFA, TGFB2, IL1A, IL6, VCAM1
Graft-versus-host disease	8	42	2.39*E*-08	HLA-B, FASLG, IL2, IFNG, HLA-A, TNF, IL1A, IL6
Type I diabetes mellitus	8	43	2.55*E*-08	HLA-B, FASLG, IL2, IFNG, HLA-A, TNF, LTA, IL1A
Inflammatory bowel disease	9	65	3.05*E*-08	IL10, TGFB1, IL2, IFNG, TNF, TGFB2, IL1A, IL6, IL4
Proteoglycans in cancer	13	205	9.11*E*-08	FLNA, HGF, FN1, CTNNB1, TGFB1, FASLG, MMP9, MMP2, TNF, VEGFA, TGFB2, PLAU, CD44
Leishmaniasis	9	76	1.04*E*-07	ITGB2, IL10, TGFB1, IFNG, TNF, TGFB2, IL1A, NOS2, IL4
African trypanosomiasis	7	36	1.44*E*-07	IL10, FASLG, IFNG, TNF, ICAM1, IL6, VCAM1
Human T cell leukemia virus 1 infection	13	222	1.84*E*-07	ITGB2, HLA-B, TGFB1, IL2, HLA-A, TNF, ICAM1, TGFB2, LTA, IL6, ITGAL, ATM, B2M
IL-17 signaling pathway	9	94	5.45*E*-07	MMP1, MMP13, CEBPB, MMP9, IFNG, TNF, MMP3, IL6, IL4
Amoebiasis	9	101	8.96*E*-07	ITGB2, IL10, FN1, TGFB1, IFNG, TNF, TGFB2, IL6, NOS2
Chagas disease	9	101	8.96*E*-07	IL10, TGFB1, FASLG, IL2, IFNG, TNF, TGFB2, IL6, NOS2
Fluid shear stress and atherosclerosis	10	139	1.17*E*-06	CTNNB1, NOS3, MMP9, IFNG, MMP2, TNF, ICAM1, VEGFA, IL1A, VCAM1
Autoimmune thyroid disease	7	52	1.39*E*-06	IL10, HLA-B, CD40LG, FASLG, IL2, HLA-A, IL4
TNF signaling pathway	9	112	1.80*E*-06	ITCH, CEBPB, MMP9, TNF, ICAM1, MMP3, LTA, IL6, VCAM1
Cell adhesion molecules	10	148	1.80*E*-06	SELP, ITGB2, HLA-B, OCLN, CD40LG, CD34, HLA-A, ICAM1, VCAM1, ITGAL
Lipid and atherosclerosis	11	214	6.35*E*-06	MMP1, SELP, CD40LG, FASLG, NOS3, MMP9, TNF, ICAM1, MMP3, IL6, VCAM1
Hematopoietic cell lineage	8	98	7.16*E*-06	KIT, ITGA1, CD34, TNF, IL1A, IL6, CD44, IL4
NF-kappa B signaling pathway	8	102	8.92*E*-06	CD40LG, EDA, TNF, ICAM1, LTA, VCAM1, PLAU, ATM
Tuberculosis	10	179	8.92*E*-06	ITGB2, IL10, CEBPB, TGFB1, IFNG, TNF, TGFB2, IL1A, IL6, NOS2

## Data Availability

The data supporting the findings of this study are available within the article.

## Supplementary Materials

Supplementary Materials Table S1: detailed gene lists related with keratoconus and allergenic disease from text mining.
