# Supplementary material for: Identification of Key Genes and Molecular Pathways in Keratoconus: Integrating Text Mining and Bioinformatics Analysis
Source: Biomed Res Int. 2022 Aug 23;2022:4740141. doi: 10.1155/2022/4740141 (PMC9427295; doi:10.1155/2022/4740141)
Supplement: Supplementary Materials — Table S1: detailed gene lists related with keratoconus and allergenic disease from text mining. [file 4740141.f1.docx]

| Table S1. Detailed gene lists related with Keratoconus and Allergic disease from text mining. | | |
| --- | --- | --- |
| Keratoconus related genes | Allergic disease related genes | Common genes |
| NID1  TBCE  TGFB2  PLXNA2  CD34  IL10  CRB1  NPL  ANGPTL1  FASLG  MYOC  SELE  SELP  BCS1L  IGFBP5  NTRK1  FN1  S100A2  S100A4  CTSK  TFPI  PHGDH  CD2  CD58  SP3  NGF  VCAM1  TNFAIP6  CHST2  PLG  ANGPTL3  IL1RN  IL1A  NOS3  AKR1A1  GJA1  TIMP2  UROD  PLK3  TAS2R38  PTPRF  TIE1  COL8A1  CD300LF  ATM  OPN1SW  VAMP8  LEP  ABO  MMP13  MMP3  MMP1  MMP10  MMP8  COL8A2  CRX  COL17A1  DMPK  FLNA  BGN  CD79A  PELI1  TGFB1  SFN  FGF4  VEGFA  MAPKAP1  CD40LG  GNPTAB  SAGE1  NGFR  CHST6  FBXO17  GSN  DCN  KERA  TNC  TNFSF15  SFTPD  SRI  PLAU  HGF  LYZ  IFNG  CTNNB1  ALK  CTSL2  LAT2  CEBPB  MMP2  PLOD1  CNOT8  DCT  TEK  VSX1  DCLRE1C  AQP3  ICAM1  VIM  SMG1  TIMP3  AQP9  TIMP1  IL6  HIST1H1T  VSX2  LOX  ITCH  FBN2  FOS  CAT  ADH1B  CD44  VASH1  ALDH3A1  AQP2  AQP5  IL4  HLA-G  HLA-A  NOS2  SPAG5  EGF  TGFBI  PDGFB  CTNNA1  IL2  RET  NUDT6  HLA-B  SP1  LTA  MATN3  TNF  MAN2C1  ITGAL  EDA  HNF4A  FGF1  SLC7A3  IGFBP3  TBC1D2B  ENO1  PSMA4  TF  GLYAT  MORF4L1  CTNNBIP1  CTSH  RASGRF1  TMED3  GLB1  MS4A2  RPS4X  IL16  MMP9  MTOR  ANGPTL7  EYA2  SLC24A3  ACTB  RBBP9  ZNF133  FBN1  COL6A1  GUCY2D  COL18A1  ITGB2  B2M  CD68  SNAP25  ANKHD1  BMP2  OCLN  KLF6  KIT  BMP4  RB1  PRNP  TFF1  TFF3  AIPL1  BCR  STS  AVP  ITGA1  GSR  DUSP4  SOD3  A2M  SIM2  PBK  PTK2B  PNMA2  SERPINF1  ENO2  ALOX5AP  SLC7A1  SMAD7  SOD1  C16orf35  TJP1  GAPDH  AQP1  BSG  MMP14  SNRPN  SLC7A2  TTR  DSG3  CTSB  AQP4  APEX1  TYMS | NME1  GSTM1  TAP2  NLRP3  CHRM3  LYST  DDT  PARP1  MIXL1  LIN9  LBR  TLR5  TGFB2  ATF3  IRF6  HSD11B1  CD34  CD46  CR1  CR2  CD55  IL24  IL10  REN  LAX1  CHI3L1  CHIT1  AGXT  RNPEP  LMOD1  NAV1  PTPRC  CSNK2B  RGS1  PTGS2  SAG  RNF2  INPP5D  NCF2  LAMC2  SOAT1  ITM2C  FASLG  CCL20  MYOC  SELL  SELP  XCL1  DES  FCGR3B  FCGR3A  FEV  CD244  CD48  CD84  SLAMF1  SLAMF6  SLC11A1  PNKD  CRP  APCS  FCER1A  IL8RA  TNS1  CD1C  HDGF  FN1  NES  LMNA  GBA  MTX1  MUC1  ICOS  CTLA4  CD28  IL6R  ALS2  SLC39A1  S100A7  S100A7A  S100A8  LOR  FLG  HSPD1  STAT4  STAT1  CTSS  MCL1  TFPI  FCGR1A  GJA8  ITGA4  CD160  TTN  PHGDH  HOXD13  CD2  CD58  SP3  NGF  SST  ADIPOQ  KNG1  PTPN22  THPO  WNT2B  GSTM3  SCN2A  GSTM2  DPP4  TNFRSF13B  RBMS1  TNFSF10  CNTF  CLDN11  VCAM1  DPYD  F3  IL12A  MBNL1  HNMT  CXCR4  CLCA1  PFN2  CPB1  CCR6  CFC1  LPA  MAS1  SOD2  FAIM  CEP70  NOX3  GYPC  IL12RB2  GLI2  MIER1  DPP10  ESR1  IL1RN  TRH  IL1B  IL1A  JUN  BCL2L11  VIPR2  B3GAT1  PTPRN2  GATA2  NLRP5  IL11  HECA  IFNGR1  FOXRED1  FCAR  FASTK  IL18R1  IL1RL1  MYLK  IL1R1  NOS3  ENPP1  ESAM  ENPP3  SLC25A10  ARG1  CD86  CFHR1  FAAH  CD80  THY1  ZAP70  ADAM8  TIMP2  PIP  HMBS  NKX6-2  PLN  SOCS3  BOC  ROS1  CD200R1  FPR2  MGMT  MKI67  SIGLEC8  CD200  NPS  HDAC2  MARCKS  WISP3  FYN  AANAT  IL10RA  TRAF3IP2  SPHK1  FOXJ1  OAT  KLK1  PTPRF  TAGLN  PTN  WASF1  PTGDS  APOA1  GRB2  NCAM1  CD8A  BAG3  IL18  C2  IRF3  CPA1  PAEP  FCGRT  FCN2  FLT3LG  TSPAN33  CD300A  ATM  SFTPB  FLNC  CASP8AP2  FOXP1  NTF4  CASP1  LEP  BAX  CNR1  ABO  MMP13  MMP3  MMP1  MMP10  MMP8  MMP7  BIRC3  KPNA2  NT5E  TMEM133  REG1A  PRKCA  CFTR  TACR1  SULT2A1  IL31  GPR83  FLNB  ORAI1  HTR1B  ICAM2  CD79B  C5AR1  GH1  ABL1  IL17RD  CTSC  IL9R  IL17RB  CHDH  NOVA2  DMPK  SLC26A3  PPRC1  SLC26A4  F8  NOS1  PRCP  PIK3CG  IKBKG  G6PD  ACE  LCK  RELB  DYSF  APOE  BCAM  EMD  SCD  PKD2L1  CD207  FLNA  OPN1MW2  OPN1MW  CPN1  KCNN4  DST  MATN1  BMP5  PSG5  CISH  SERPINE1  MPO  ABCD1  EPX  ZMYND10  CIC  PTAFR  IL17F  IL17A  DPEP1  COIL  CD79A  EPO  CDK9  FGR  CFB  UGP2  SELPLG  IL18BP  TGFB1  CYBA  GABRQ  DNTT  AC005921.3  NR0B2  FGF3  CD52  VEGFA  MAPKAP1  TRIM63  REL  CDR1  MBTPS1  AMT  RHOA  FANCL  USP4  F9  PAH  IGF1  GSTP1  CD40LG  GNPTAB  CYP3A4  CLC  NGFR  CPSF4  ADRBK1  GRHL3  IL28RA  SLC25A20  APAF1  FAS  NFKBIB  SP6  ASNS  TAC1  GSN  TBX21  HP  DHODH  UCN2  ITGB3  ACTN4  PON1  WAPAL  ODZ1  CDK5RAP2  MDFI  TLR4  EPCAM  TREM1  CRHR1  HSPG2  CAMP  TNC  TNFSF8  KITLG  SFTPD  GFAP  DNAH8  NTS  COG8  SRI  CDH1  PIM1  HAAO  ABCB1  CDKN1A  LTF  NBL1  CCR5  CCR1  CCR3  CXCR6  MAPK14  ACD  IL13RA2  RNF130  PLAU  HGF  FKBP5  LTC4S  CD36  BRCA1  PPARD  TXN  AOC3  NOL3  LYZ  ABHD5  CPM  IKBKAP  RRAD  CCR10  CD22  MAG  DDX41  USF2  MLN  IL22  IL26  IFNG  AC132186.2  TH1L  STAT3  SMC2  VIPR1  BIRC6  CCL24  CCL26  PLP1  F11  TAPBP  XDH  CTNNB1  TLR3  PRF1  NODAL  HRH2  ALK  BMP7  CCR8  SLC25A4  EIF1  KRT17  TACR2  HLA-DPB1  FURIN  ARSA  CCL17  CX3CL1  CASP3  CCL22  GTF2I  BRD2  SCN11A  IQGAP1  TAP1  GLA  SCN5A  TYMP  LAT2  HERPUD1  DDIT3  AGA  ANPEP  BTK  CCR7  HLA-DQA1  MT1A  MT1E  HLA-DRB1  ELN  CEBPB  MYD88  IL12B  TNFRSF1B  CSF3  MMP2  TNFRSF8  STAT6  SCRT1  SH3RF1  SCGB1A1  ORMDL3  ITK  HAVCR2  HAVCR1  UCN  GRB7  SGCD  ERBB2  NPPA  FEN1  CYLD  NOD2  CDCA4  CYSLTR1  SDAD1  SEC14L3  CXCL9  CXCL10  CXCL11  PDE7A  PIK3C2A  CYP19A1  CRH  FOXA2  IFNB1  ARG2  CYBB  IFNA5  IFNA2  IFNA1  CDKN2A  EDN1  SELM  ANTXR2  MUC16  IFNK  CD83  LY75  IL5RA  PMP22  RTEL1  HM13  TPMT  BAG1  SLCO6A1  HPSE  BCL2L1  LY96  PRL  ICAM1  VIM  IL7  SYN3  SMG1  AHR  ICAM3  TYK2  AGT  ADAM10  TSLP  FABP4  PDE4A  MRC1  APC  MCC  AFF1  HMOX1  BDNF  TNFSF13B  MB  DNAI1  VDR  TIMP1  SPP1  COL2A1  IL6  HIST1H1T  PLUNC  CCL27  MYH9  CCL21  RUNX1T1  PGDS  WAS  SLC12A2  ITCH  FBN2  KIAA0101  TST  CAT  IL2RB  SPG21  TGFB3  SIT1  CD44  IL3  CSF2  GSTT1  ALDH3A2  GSTZ1  NFKB1  IRF1  IL5  MOG  RAD50  LGALS1  HRH1  ALKBH1  IL13  IL4  HLA-A  ACP1  TNFRSF4  NOD1  PPARG  FOXP3  BAMBI  LGALS9  TPO  SMAD6  NOS2  HSPA4  SMAD3  IL4R  IL21R  OSTC  HLA-E  VTN  ADI1  CFI  EGF  IL9  SCN8A  NPSR1  IL27  ITGB1  NR4A1  TSPYL2  AOAH  ADAM17  IL27RA  CD19  HEXA  KRT1  LAT  PTGER1  KRT8P9  COLQ  LGMN  FGD1  CD276  TGM2  LOXL1  COL14A1  HBEGF  IL2  BTD  IL21  FGF2  NUDT6  HLA-C  INTU  HLA-B  CXCL12  ARID3B  CD14  ALOX5  CYP1A1  MAPK3  SERPINA1  ANXA1  SP1  RAB5A  NDUFB9  MTSS1  SERPINA3  LTA  TNF  RPL22  MYC  NDUFA6  CYP2D6  TCL1A  ITGAL  BDKRB2  IL15  MYBL2  SMUG1  CCL2  APOB  EDA  CCL11  CCL13  PRG2  CCL1  CSPG4  GYPE  GYPB  TG  MIF  ADA  SERPING1  ADCY1  MAPK8  IGFBP1  SMAD1  IGFBP3  LARS  WARS  SLPI  EDNRA  ENO1  CCL5  CLIC1  CCL15  EIF2C2  CCL18  CCL3  PTK2  CCL4  POMC  TLR9  SPINK5  TF  CD63  ASL  ADRB2  HSP90AA2  ITGAM  ITGAX  HSPA1L  HSPA1A  GIF  HSPA1B  JAK3  CCR4  GLB1  NEU1  CXCR3  MS4A2  EGFR  RPS4X  CTSL1  TLR2  IL12RB1  PDGFRB  IL16  MS4A1  MMP9  MBL2  CD40  GPR44  PPARA  ABCB7  SYK  GPX3  GUSB  IL23A  AKT1  GLS2  MTHFR  OPTN  MAX  CALCA  HDC  SOCS1  EDNRB  AREGB  AREG  ACTB  SSR1  EPGN  ESR2  PTH  CXCL5  PPBP  PF4  FCER2  CXCL1  IL8  RETN  PLAG1  NF2  PER1  AFP  LYN  SAT1  GATA3  PDXP  TBCA  ALB  DDX53  PHEX  KIN  F2RL1  SMS  RRBP1  PTHLH  PPL  ITGB2  MN1  HTN1  IL2RA  B2M  ZNF143  SRL  TP53  C3  IL33  DNASE1  CD68  JAG1  SNAP25  PDCD1LG2  MEFV  JAK2  NHS  TUB  IKBKB  GGT1  PLAT  BMP2  SLC1A1  LGALS3  OCLN  OR10A4  GCH1  OR2AG1  KIT  PDGFRA  PHF11  SETDB2  IDO1  FANCB  PCNA  CD180  PTGDR  ITM2B  ADRA1D  TMSB4X  TLR8  TLR7  TFF2  GTPBP4  IL13RA1  AC055839.1  BCR  MGP  C1QBP  KAL1  EMP1  TPT1  UCHL1  MAPK1  ADAM33  STS  ADRB3  IL6ST  NFATC1  DDX4  DAPK3  MBP  GZMA  IL3RA  GZMK  TLR6  TLR1  TYRO3  CSF2RA  ITGA2  ITGA1  NRG1  TBXA2R  RBPSUH  RBPMS  NFKBIA  POSTN  CD69  SNRPB  KLRB1  FAM48A  PDYN  SERPINB3  EXTL3  A2M  ITGAE  BCL2  AICDA  ESCO2  TRPV1  INS  C3AR1  TPSAB1  CD38  HLA-DRB3  ADCY10  GZMB  CMA1  CLNK  THBS1  PNMA2  IL7R  BRCA2  DUSP8  GNRH1  NEFM  LTB4R  SLC25A1  STUB1  MUC2  PTPN6  C12orf57  SERPINF2  SEC14L2  ENO2  ALOX5AP  TCF3  SLC45A2  TPI1  TNFRSF10A  SMAD4  TNFRSF10D  C15orf55  BID  CD4  FCGR2B  NRL  SLC39A14  CYTL1  SMAD7  CHRNA7  SMAD2  BMP1  MRAP  TRIO  SOD1  CHRFAM7A  MPG  IL25  GAPDH  CFD  PRTN3  SETBP1  CD27  TNFRSF1A  PALM  IRX2  NAT2  NAT1  CD9  IL17D  VWF  APP  NTF3  BTG3  PDE6B  CTSB  HRH4  IMPACT  RNASE2  RNASE3  NPC1  MIB1  DEFA5  DEFA6  DEFB1  ANGPT2  EPB41L3  AC217770.1  AF347015.27 | TGFB2  CD34  IL10  FASLG  MYOC  SELP  FN1  TFPI  PHGDH  CD2  CD58  SP3  NGF  VCAM1  IL1RN  IL1A  NOS3  TIMP2  PTPRF  ATM  LEP  ABO  MMP13  MMP3  MMP1  MMP10  MMP8  DMPK  FLNA  CD79A  TGFB1  VEGFA  MAPKAP1  CD40LG  GNPTAB  NGFR  GSN  TNC  SFTPD  SRI  PLAU  HGF  LYZ  IFNG  CTNNB1  ALK  LAT2  CEBPB  MMP2  ICAM1  VIM  SMG1  TIMP1  IL6  HIST1H1T  ITCH  FBN2  CAT  CD44  IL4  HLA-A  NOS2  EGF  IL2  NUDT6  HLA-B  SP1  LTA  TNF  ITGAL  EDA  IGFBP3  ENO1  TF  GLB1  MS4A2  RPS4X  IL16  MMP9  ACTB  ITGB2  B2M  CD68  SNAP25  BMP2  OCLN  KIT  BCR  STS  ITGA1  A2M  PNMA2  ENO2  ALOX5AP  SMAD7  SOD1  GAPDH  CTSB |
